# Rapid, simple and inexpensive production of custom 3D printed equipment for large-volume fluorescence microscopy

**DOI:** 10.1016/j.ijpharm.2015.03.042

**Published:** 2015-10-30

**Authors:** Adam L. Tyson, Stephen T. Hilton, Laura C. Andreae

**Affiliations:** a MRC Centre for Developmental Neurobiology, King’s College London, London SE1 1UL, UK; b Department of Forensic and Neurodevelopmental Science, King’s College London, London SE5 8AF, UK; c Department of Pharmaceutical and Biological Chemistry, UCL School of Pharmacy, University College London, London WC1N 1AX, UK

**Keywords:** ABS, acrylonitrile butadiene styrene, PLA, polylactic acid, FDM, fused deposition modelling, SDS, sodium dodecyl sulphate, PFA, paraformaldehyde, STL, stereolithography, 3D printing, Additive manufacturing, Optical clearing, CLARITY

## Abstract

The cost of 3D printing has reduced dramatically over the last few years and is now within reach of many scientific laboratories. This work presents an example of how 3D printing can be applied to the development of custom laboratory equipment that is specifically adapted for use with the novel brain tissue clearing technique, CLARITY. A simple, freely available online software tool was used, along with consumer-grade equipment, to produce a brain slicing chamber and a combined antibody staining and imaging chamber. Using standard 3D printers we were able to produce research-grade parts in an iterative manner at a fraction of the cost of commercial equipment. 3D printing provides a reproducible, flexible, simple and cost-effective method for researchers to produce the equipment needed to quickly adopt new methods.

## Graphical abstract

**Figure:**
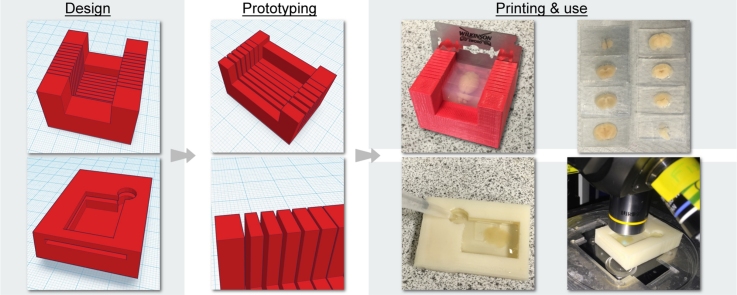

## 1 Introduction

As research questions in neuroscience, and biomedical science in general, become more complex, so too do the techniques involved. Methodological advances also appear more frequently, leading to a greater rush for research groups to utilise these within their field. However, each novel method brings its own problems in the equipment that is required. A good example of this is tissue clearing (Höckendorf et al., 2014, Kim et al., 2013), the process by which biological tissue is rendered transparent to allow for light microscopic investigation of large volumes of brain tissue. A number of tissue-clearing methods have been recently developed (Chung et al., 2013, Hama et al., 2011, Ke et al., 2013, Kuwajima et al., 2013, Susaki et al., 2014), but the common factor amongst them is that the volumes of tissue involved are orders of magnitude greater than traditional histology, requiring custom laboratory equipment for both the handling and imaging of samples.

Laboratory equipment for these novel techniques can be produced in a number of ways. Commercial manufacturers will develop equipment once experimental techniques have become commonplace, but this is often too late for those researchers wishing to quickly adopt a method and are frequently prohibitively expensive. In addition, these products are often “generic” in nature, so they may fail to precisely provide the desired function, delaying further innovation. Alternatively, custom equipment can be constructed using conventional manufacturing techniques: additive (e.g. welding), net shape (e.g. injection moulding) and subtractive (e.g. machining). However, these methods are expensive and require specialist equipment and training, which is beyond many research groups. External collaborators or contractors may provide this equipment and knowledge, but this can be at the expense of speed, which is required for the rapid prototyping of custom parts.

Layered, additive manufacturing (3D printing) overcomes these problems, allowing for rapid, simple and inexpensive prototyping of custom parts for research. The concept has existed for some time (Hull, 1986), but until recently it has remained expensive and complicated, as is often the case with manufacturing techniques. One method of 3D printing is fused deposition modelling (FDM), (Crump, 1989), which has seen a dramatic decrease in the cost of individual printers and is now readily available to the consumer market. FDM printers are available in either kit form or fully assembled for between $300 and $5000 depending on the specifications of the machine. These printers work by melting a plastic filament and depositing a layer of material onto a moveable platform. The platform then moves vertically away from the printing head to allow the next layer to be deposited onto the existing layers, allowing a 3D object to be generated. In addition to the low cost, while previously 3D printing required in-depth computer aided design knowledge, most simple parts can now be developed with simple and freely-available tools.

The aim of this study was to explore the use of freely-available software, along with inexpensive consumer grade FDM printers, to produce custom equipment required for a novel tissue clearing method: passive CLARITY (Chung et al., 2013, Tomer et al., 2014).

## 2 Materials and methods

### 2.1 Materials

Polylactic acid (PLA) and acrylonitrile butadiene styrene (ABS) filaments were purchased from 3D FilaPrint (UK); 40% acrylamide and 2% bis-acrylamide solutions from Bio-Rad (UK); 16% paraformaldehyde (PFA) solution from Alfa Aesar (UK); VA-044 photoinitiator from Wako Chemicals (Germany); agarose from Roche (UK); sodium dodecyl sulfate (SDS), boric acid and sodium hydroxide (NaOH) from Sigma–Aldrich (UK); phosphate buffered saline (PBS) tablets and glycerol from Fisher Scientific (UK).

### 2.2 Design software

All models were designed using Tinkercad software (www.tinkercad.com) and exported as STL (STereoLithography) files to MakerBot Desktop printers for printing. Final versions of the STL files are available as Supplementary materials.

Supplementary material related to this article found, in the online version, at http://dx.doi.org/10.1016/j.ijpharm.2015.03.042.

### 2.3 3D printing

All models were produced using either a MakerBot Replicator Mini (using PLA) or a MakerBot Replicator 2X (using ABS) purchased from MakerBot Industries, LLC. All printing parameters are shown in Table 1. Vernier callipers were used to verify the model dimensions after printing.

### 2.4 Clearing

Adult (C57BL/6) mice were perfused with PBS and brain tissue was fixed with 4% PFA for 24 h. Following this, whole brains were prepared according to the passive CLARITY protocol (Tomer et al., 2014). Each brain was incubated in 40 ml of hydrogel solution (4% acrylamide, 0.05% bis-acrylamide, 0.25% VA-044 and 4% PFA in PBS) at 4 °C for 10 days. The hydrogel was degassed using a vacuum pump, a desiccation chamber and nitrogen gas before polymerisation at 37 °C for 2 h. The brain was removed from the excess hydrogel and embedded in a 6% agarose solution for sectioning. After sectioning, the tissue was cleared in a 4% SDS solution in sodium borate buffer (pH 8.5 with NaOH). Tissue sections were cleared at 37 °C with shaking, and the clearing buffer was replaced weekly until the slice could be placed over printed text without visible distortion of the letters. Prior to imaging, samples were incubated in refractive index (RI) matching solution (85% glycerol) for 24 h.

## 3 Results and discussion

### 3.1 Brain slicing matrix

To achieve optimal tissue clearance and antibody staining using the passive CLARITY protocol (Tomer et al., 2014) mouse brain tissue is sectioned before clearing. Sectioning also complements the use of more readily-available microscopy methods (confocal and multiphoton imaging, rather than single-plane illumination microscopy). Traditional rodent-brain matrices for sectioning tissue are commercially available, but these are expensive (∼$200 for a single acrylic matrix and $350–$600 for a stainless steel matrix). Further, each matrix is designed for brains of a defined species and age, which significantly limits the flexibility of their use. They are also difficult to use unless the tissue aligns perfectly with the matrix. CLARITY renders tissue very soft and difficult to handle, so agarose embedding prior to sectioning and clearance is desirable. Tissue matrices designed to slice blocks of embedded tissue are also available, but these are equally expensive (∼$400) and inflexible.

We therefore aimed to produce a simple design that could be used to section brain tissue in a reliable and reproducible manner, and which could be rapidly adapted to changing experimental design. It was also important that the matrix could be designed using freely available software and printed using a consumer grade FDM printer.

**Fig. 1:**
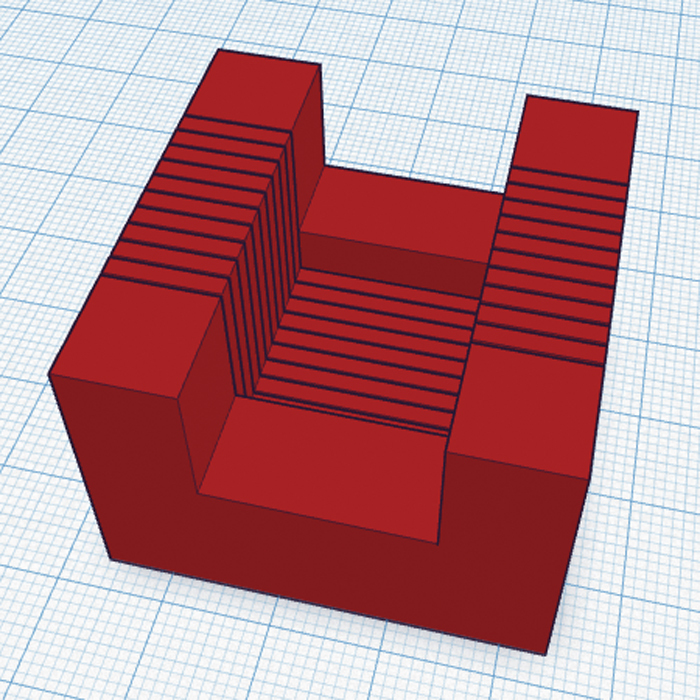
Initial brain slicing matrix design.

Of the available 3D-design tools which can be used with FDM, we chose Tinkercad. This is a free online tool that allows extremely simple 3D design for non-specialists. Rather than adopting a complex 3D approach, a number of pre-defined shapes can be easily combined into the final design. Fig. 1 shows the initial design for the brain slicing matrix. This shape would allow an agarose embedded brain to be accurately sectioned into 2-mm-thick coronal slices using a razor blade, which could then be easily removed from the matrix without damaging the tissue.

**Fig. 2:**
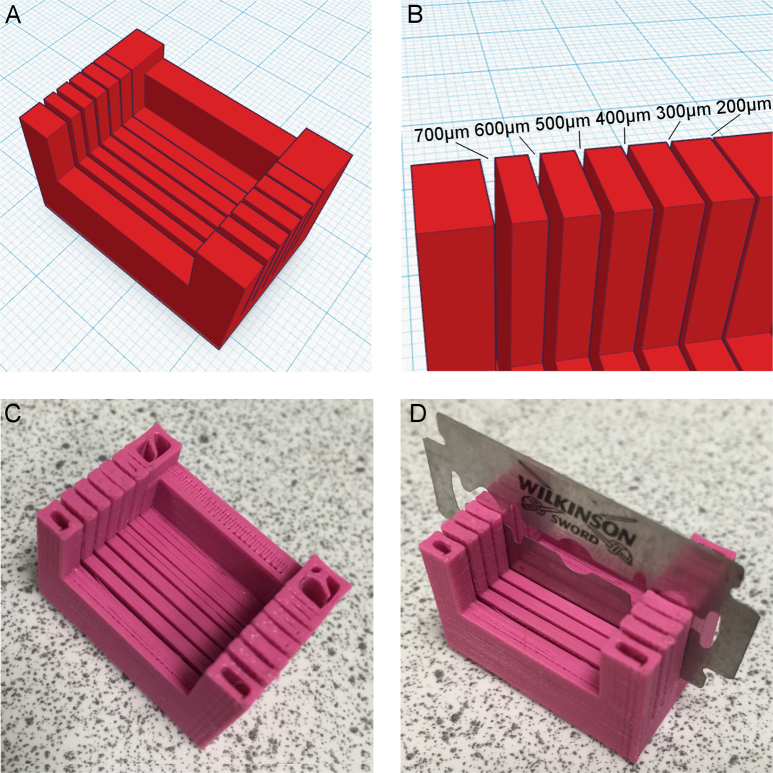
Optimisation of slit width within a single design. (A) Model incorporating six potential slit widths. (B) Slit widths between 200 μM and 700 μM. (C) PLA print of the model. (D) Determining the optimal slit width: razor blade fits within the optimal width (400 μM).

One of the limitations of consumer grade FDM printers is their spatial resolution. The nature of fused deposition means that there can be ‘blurring’ of the print when the printing nozzle moves the plastic from its precise, intended location. This can lead to very fine details being obscured and small gaps being filled in. The “slits” in the design in Fig. 1 need to be exactly 2 mm apart for precise sectioning. However, the width of the slits themselves needs to be sufficiently wide to allow a razor blade to fit into them, but narrow enough to keep the blade perpendicular to the tissue surface, allowing for accurate and reproducible sectioning. To quickly determine the slit width needed, another model (Fig. 2A) was produced. This model was simply a modification of the original design in which the slit widths varied between 200 μM and 700 μM at 100 μM intervals (Fig. 2B). The relative ease and speed of 3D printing, coupled with the use of inexpensive filaments, meant that it was simple to rapidly optimise designs, including (as here) in a single print.

The model in Fig. 2A was printed on both a MakerBot Replicator Mini (using PLA) and a MakerBot Replicator 2X (using ABS), both inexpensive consumer grade FDM printers. The optimal design was printed on the MakerBot Replicator Mini using PLA. Final 3D printing parameters are outlined in Table 1.

**Fig. 3:**
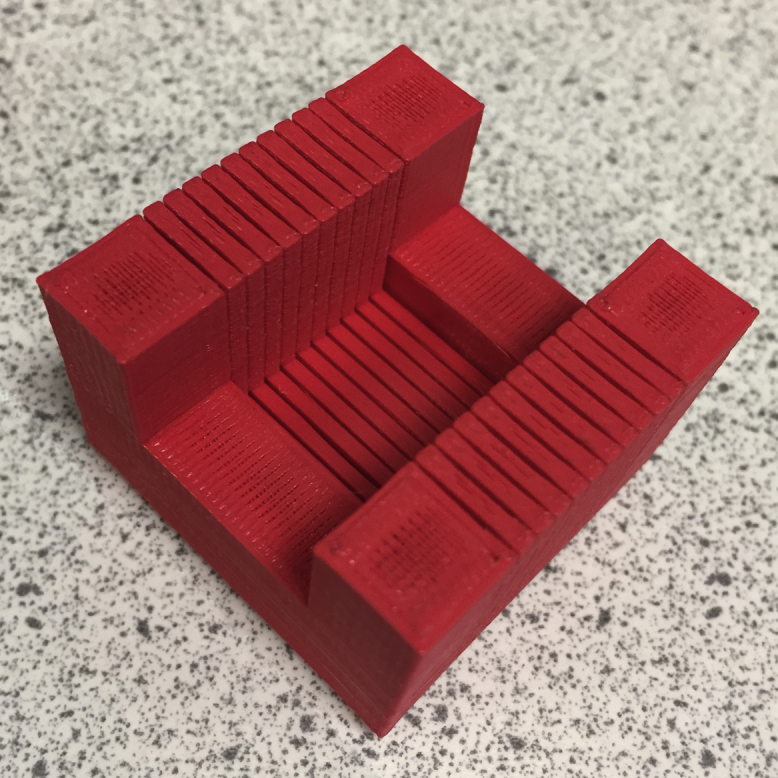
Final PLA print of brain slicing matrix with 400 μM slits.

**Fig. 4:**
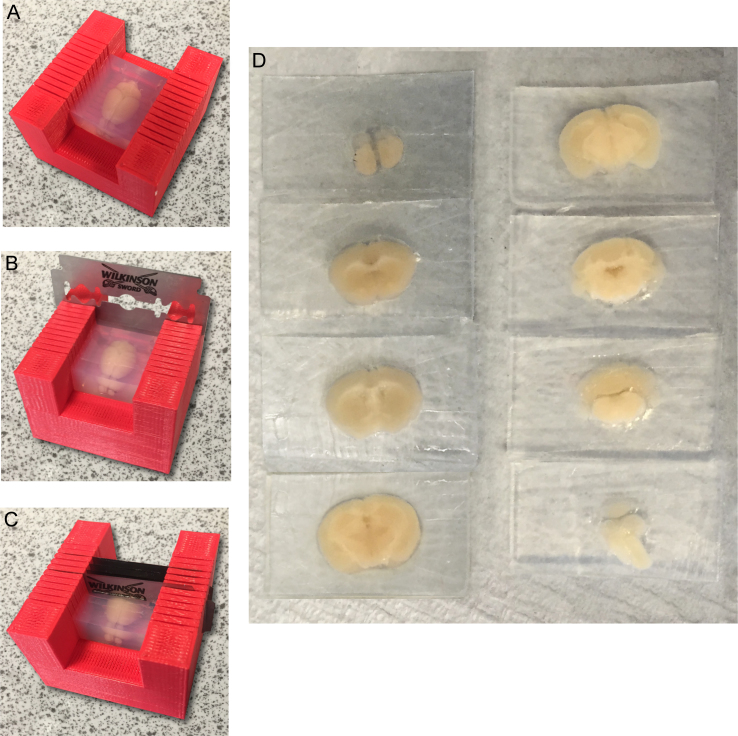
Application of brain slicing matrix. (A–C) Using the brain slicing matrix to generate coronal sections of an adult mouse brain embedded in agarose. (D) 2 mm coronal mouse brain slices, ready for clearing using CLARITY.

Fig. 2C shows the physical print of Fig. 2A in PLA from the MakerBot Replicator Mini. Fig. 2D shows the model with a razor blade in the 400 μM slit, which was the optimal thickness for the final print. The final print is shown in Fig. 3. This model was then successfully used to section an agarose-embedded mouse brain into accurate 2 mm slices (Fig. 4) ready to be cleared.

### 3.2 Staining and imaging chamber

**Fig. 5:**
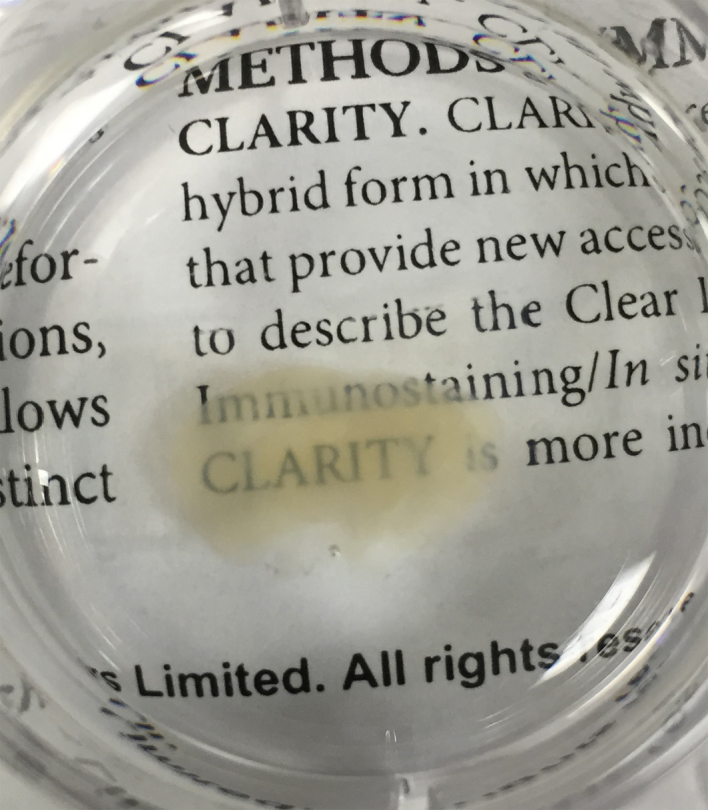
2 mm coronal mouse brain section following clearing with CLARITY.

In CLARITY, following sectioning and clearing, the tissue can be antibody stained and imaged. This presents two problems. First, the cleared tissue (Fig. 5) is very fragile, and moving it between containers can potentially damage it. Second, traditional sample-mounting set-ups (a glass slide and coverslip) are not well designed for the imaging of large volumes of tissue for extended periods of time. To overcome these issues, a combined staining and imaging chamber was designed. The goal was to ensure that the cleared tissue sections could be transferred to this container, allowing staining, washing, matching and imaging to then be performed without handling the sample.

**Fig. 6:**
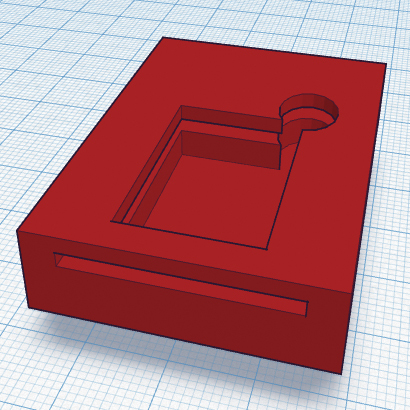
Combined staining/imaging chamber design.

**Fig. 7:**
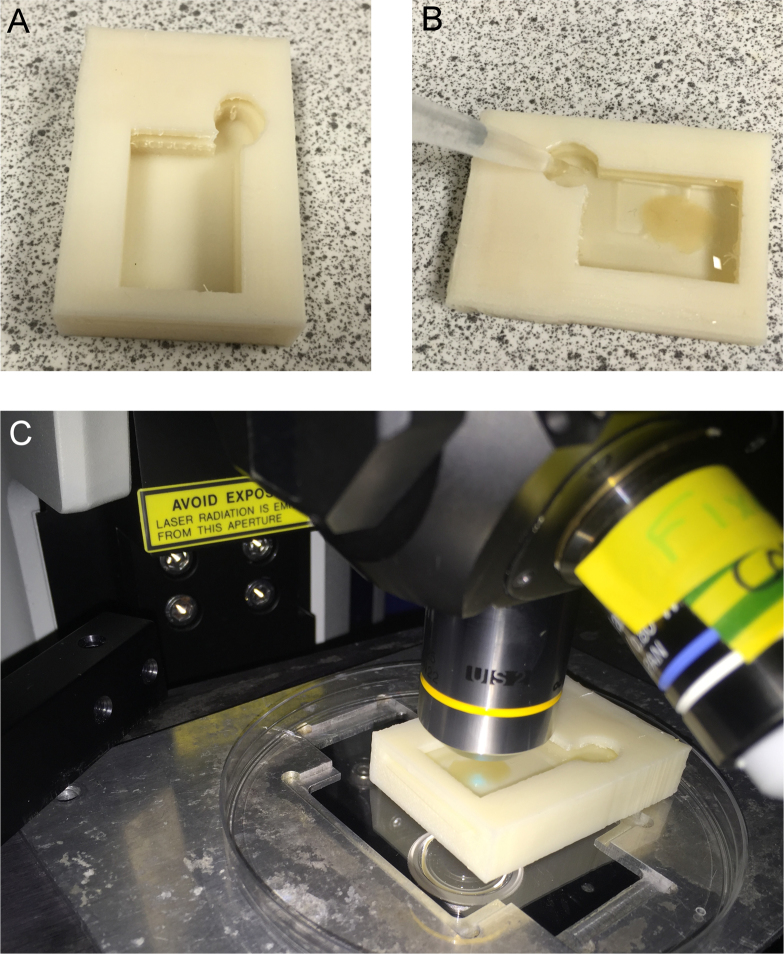
Staining/imaging chamber. (A) ABS print of the combined staining/imaging chamber. (B) Filling the chamber with antibody solution for staining. (C) Confocal imaging of a cleared slice in the chamber.

A chamber was designed (Fig. 6) for this purpose, including a filling/aspiration port to prevent contact with the sample. The chamber was sized to fit tissue which had been sectioned using the 3D printed matrix shown in Fig. 3. In addition, the design incorporated a small slit in the top for insertion of a standard 22 × 40 mm coverslip, allowing imaging within the chamber on a conventional upright confocal or multiphoton microscope. The model was printed vertically (on the smallest end) and with removable supports to allow the overhangs to be produced. The Replicator 2X was used with ABS, as ABS is more resistant to degradation than PLA (necessary for long antibody incubations). The final print is shown in Fig. 7A. This chamber can then be used for staining, washing and RI matching (Fig. 7B) as well as for microscopy (Fig. 7C). The custom size of the chamber also helps to reduce the amount of expensive antibody solution and RI matching solution required to the minimum necessary.

## 4 Conclusion

The present study shows how custom laboratory equipment can be designed, tested and built easily and simply using freely available software, and consumer grade 3D printing equipment. This application to a novel tissue clearing method (passive CLARITY) is just one of many within biomedical science. Applications to both magnetic resonance imaging (Herrmann et al., 2014) and in vitro electrophysiology (Hyde et al., 2014) have been published previously.

While 3D printing has been available for some time, it is only very recently that the technology could be readily adopted by most laboratories. The software used in this study is very simple, and can be employed to design a wide variety of parts after only a few minutes of training. The printing software is also very easy to use, and works with commercially available printers. In addition, consumer-grade printers require far less maintenance than self-assembled machines. For example, exchangeable filament-extrusion heads such as those available with the MakerBot Replicator Mini’s reduce printer downtime due to blocked extruders. Researchers can therefore devote their time to the research question at hand rather than making parts or maintaining equipment.

One of the most important aspects of any scientific study is reproducibility. 3D printing provides this both within a study, and for replication. Once a part has been designed, any number of copies can be made to allow for a consistent experimental set-up. Once a study has been published, both the model design (STL file) and the printing parameters can be made freely available, allowing anyone to reproduce the work using exactly the same equipment.

Another key benefit of 3D printing is the flexibility it allows. Commercial products are extremely inflexible, and traditional manufacturing requires a great deal of time, effort and money to adapt designs. The models used in this study are good examples. If the optimal slice thickness for CLARITY was to be changed, it would be trivial to adapt the brain matrix to slice at a different thickness, and to change the staining/imaging chamber to accommodate a different size slice. Similarly, should it become necessary to dissect a particular brain area for analysis (as opposed to simple slices), a brain atlas could be used to design a chamber to allow for accurate and reproducible dissection of specific brain regions very easily.

Lastly, the main benefit of 3D printing, particularly the approach used in this study, is the cost. 3D printers require an initial outlay, but this cost is reducing each year. The printers used in this study retail at approximately $1500 (MakerBot Replicator Mini) and $3000 (MakerBot Replicator 2X) in the UK. While this cost is not trivial, it compares very well to laboratory equipment in general. The running costs of the machines, however, are extremely low. One kilogram of PLA or ABS filament can be purchased for under $30, and so even with prototyping, each of the parts printed in this study cost less than $1. Compared to $170–$650 for a commercially produced matrix, 3D printers are extremely cost-effective for producing equipment and are now a viable option for most research groups.

## Figures and Tables

**Table 1:** 3D printing parameters.

Model	Brain slicing matrix	Staining/imaging chamber
Printer	MakerBot Replicator Mini	MakerBot Replicator 2X
Filament	PLA	ABS
Supports	No	Yes
Raft	Yes	Yes
Infill (%)	10	15
Shells	2	2
Layer height (mm)	0.2	0.1
Extruder temperature (°C)	230	230
Build plate temperature (°C)	N/A	120
Extruder speed while extruding (mm/s)	90	90
Extruder speed while travelling (mm/s)	150	150
Model weight (g)	24.55	12.19
Model cost ($, approx.)	0.69	0.34
Print time (hours, approx.)	3	2
